# Promiscuous prediction and conservancy analysis of CTL binding epitopes of HCV 3a viral proteome from Punjab Pakistan: an In *Silico *Approach

**DOI:** 10.1186/1743-422X-8-55

**Published:** 2011-02-08

**Authors:** Abida Shehzadi, Shahid ur Rehman, Muhammad Idrees

**Affiliations:** 1 Bioinformatics Division, Centre of Excellence in Molecular Biology, University of the Punjab, Lahore, Pakistan; 2 Division of Molecular Virology, Centre of Excellence in Molecular Biology, University of the Punjab, Lahore, Pakistan

## Abstract

**Background:**

HCV is a positive sense RNA virus affecting approximately 180 million people world wide and about 10 million Pakistani populations. HCV genotype 3a is the major cause of infection in Pakistani population. One of the major problems of HCV infection especially in the developing countries that limits the limits the antiviral therapy is the long term treatment, high dosage and side effects. Studies of antigenic epitopes of viral sequences of a specific origin can provide an effective way to overcome the mutation rate and to determine the promiscuous binders to be used for epitope based subunit vaccine design. An *in silico *approach was applied for the analysis of entire HCV proteome of Pakistani origin, aimed to identify the viral epitopes and their conservancy in HCV genotypes 1, 2 and 3 of diverse origin.

**Results:**

Immunoinformatic tools were applied for the predictive analysis of HCV 3a antigenic epitopes of Pakistani origin. All the predicted epitopes were then subjected for their conservancy analysis in HCV genotypes 1, 2 and 3 of diverse origin (worldwide). Using freely available web servers, 150 MHC II epitopes were predicted as promiscuous binders against 51 subjected alleles. E2 protein represented the 20% of all the predicted MHC II epitopes. 75.33% of the predicted MHC II epitopes were (77-100%) conserve in genotype 3; 47.33% and 40.66% in genotype 1 and 2 respectively. 69 MHC I epitopes were predicted as promiscuous binders against 47 subjected alleles. NS4b represented 26% of all the MHC I predicted epitopes. Significantly higher epitope conservancy was represented by genotype 3 i.e. 78.26% and 21.05% for genotype 1 and 2.

**Conclusions:**

The study revealed comprehensive catalogue of potential HCV derived CTL epitopes from viral proteome of Pakistan origin. A considerable number of predicted epitopes were found to be conserved in different HCV genotype. However, the number of conserved epitopes in HCV genotype 3 was significantly higher in contrast to its conservancy in HCV genotype 1 and 2. Despite of the lower conservancy in genotype 1 and 2, all the predicted epitopes have important implications in diagnostics as well as CTL-based rational vaccine design, effective for most population of the world and especially the Pakistani Population.

## Background

Family Flaviviridae comprises small enveloped pathogens classified in three genera: *Flavivirus*, *Pestivirus*, and *Hepacivirus*. Members of these genera cause various diseases in humans and other animals such as birds, horses and pigs. The only genera *Flavivirus *contain more than 70 members including Hepatitis C Virus (HCV), Dengue virus, West Nile virus and tick-borne encephalitis virus [1-3].

HCV is a positive sense RNA virus affecting approximately 180 million people world wide and rate of infection in Pakistani population is about 10 million [4,5]. HCV genome contributes about 9400 nucleotides that encode single polyprotein of approximately 3010 to 3033 amino acids in length [6]. This single polyprotein is processed by viral as well as host proteases into three structural proteins (i.e. core, E1 and E2) and four non-structural proteins (i.e. NS2, NS3, NS4, and NS5A) [7].

HCV mainly spreads via blood supply, reuse of glass syringes and needles, unsterilized medical equipment, use of tooth brushes of HCV patients, etc [7] and causes of acute and chronic infections [8]. Clinical demonstrations of acute Hepatitus C Viral infection include Jaundice, Fever, Myalgia, Fatigue, Lethargy, Increased ALT, Anorexia and Fulminant hepatic failure [7]. About 80% of HCV infected individuals develop chronic infections [9]. Chronic liver infections develop chronic hepatitis, cirrhosis and hepatocellular carcinoma within a period of 10, 20 and 30 years respectively followed by viral infection [10,11]. Out of 70-80% chronically infected individuals, 20% develop cirrhosis and 1-5% individuals suffer from final stage of liver diseases [12]. Hepatic steatosis is the accumulation of lipids in hepatocytes and is reported for the cause of cirrhosis [13] with the more severe cases being reported in patients infected with HCV genotype 3a [14]. The prevelance of steatosis in Pakistani population is about 61.5-65.5% compared with 32.8-81.2% in western countries [15]. The percentage of males infected with HCV chronic liver stage is higher then females with the age of patients between 40-50 years [5].

HCV is classified into six genotypes each heaving various subtypes [16-18]. These genotypes are distributed differently in various parts of the world with the genetic variance between them is about one third. The genotypes 1, 2 and 3 have world wide distribution. But the significant differences are observed in subtype distribution. Subtype 1a is mostly found in North America and Europe followed by 2b and 3a. Subtype 1b is frequently found in South East Europe and Tunisia and 2c in North Italy. Genotype 4 is mainly restricted to Middle East and Central Africa and genotype 5 in South Africa. Genotype 6 is distributed throughout South East Asia and also being isolated from Hong Kong and Vietnam [17]. The most frequent HCV genotypic distribution in Pakistan is 3a [49.05%] followed by 3b [17.66%] [19]. The knowledge of HCV distribution is crucial for treatment therapy and vaccination because of its predictive value in terms of response to antiviral therapy and vaccination. Effective responses to antiviral therapy are normally associated with genotype 2 and 3 in comparison to any other genotype [20].

HCV replicates at about 10^12 ^new HCV viruses/day. Replication is carried out by RNA dependent RNA polymerase. RNA polymerase lacks the "proofreading" ability that ensures the high mutation rate of about 8-18 mutations in genomic RNA/year [21,20]. Such a high mutation rate limits the treatment therapy and vaccination. The current treatment therapy for HCV is INF alpha along with ribavirin limited to about 50% population [22]. Although the response rate is not much deterring, but high dosage, long-term treatment and side effects limits the usage [23,21]. There is the possibility that after next few years, new antiviral agents such as inhibitors of the viral protease, helices or polymerase will further improve the response rate of the current therapeutic agents. However, antiviral therapy is not affordable in most developing countries, where the prevalence of HCV is generally the highest. Thus, given the huge reservoir of HCV worldwide, the development of an effective vaccine may be the cheapest way to control disease associated with HCV infection.

Development of an effective HCV vaccine requires understanding of immune response. Viral immune response is associated with Major Histocompatabiliy complex protein (MHC) and T lymphocytes/T cell. MHC are classified into 2 broad categories, MHC I and MHC II [24]. MHC initially recognizes the viral antigenic epitopes and presents to T lymphocytes for degradation. MHC I presents the antigenic epitopes to CD8+ T cells and MHC II presents to CD4+ T cells for viral degradation [25,26]. CD8 T cells also referred to as cytotoxic T cells (CTL or Tc), limit viral infections by initial recognizing and their subsequent killing infected cells and secreting cytokines. CD4 T referred to as helper cells or Th cells and provides growth factors and signals for generation and maintenance of CD8 T cells [27]. T cells recognize the antigens only when they are associated with MHC, surface glycoprotein exposed on surface of all vertebrate cells. The selection of T cell epitopes is also important because these are linear and hence easy to synthesize.

A particular vaccine developed against HCV can't be effective for Pakistani population because of variations in HCV genomic sequences and distribution with regard to geographical area. Since a large number of Pakistani population is infected by HCV3a and number of patients enrolled in public and hospitals is increasing day by day. So there is a current need to develop a vaccine against HCV in particular to HCV3a that will cover approximately maximum Pakistani population. The current vaccines are DNA vaccine, Peptide vaccine and epitopic vaccines. Epitopes are the small antigenic segments of viral proteins and causes infections in the host. Epitopic vaccines provide more potent and controlled immune response and eliminates the potential lethal effects of the use of whole viral proteins [28]. Promiscuous epitopes (epitopes capable of binding maximum number of HLA alleles) may overcome the population coverage. Secondly the conserved epitopes reduces antigen escape associated with the viral mutation [29]. So the present study was designed for the prediction of promiscuous epitopes and to analyze their conservancy in general population. Any mutation in the peptide/epitope will lower the conservancy, so it was hypothesized to analyze the pI value of the mutated amino acid residue, that if remain in the range as was in original epitope provides the likeliness of that particular epitope to be used for epitopic vaccine design having an effective control over viral mutation, immune response with minimum side effects.

## Methods

### Sequence Retrieval and Analysis

The sequence of fully sequenced HCV 3a genome and protein of Pakistani origin was retrieved from NCBI [GU294484]. The number of individual bases in the genome i.e. the number of adenine; cytosine, guanine and thymine were calculated from DDBJ database. The molecular weight of proteins, percentage of highly repeated amino acid and the least repeated amino acid in the viral protein was calculated by using sequence and search analysis tool at PIR database (http://pir.georgetown.edu/).

### Epitope Prediction

Promiscuous epitopes of HCV 3a viral proteins were predicted for HLA I and HLA II binding alleles using freely available immunoinformatics tools such as ProPred I, and ProPred respectively. In comparison to other epitope prediction tools, Propred 1 and Propred cover maximum number of Human Leukocyte antigens i.e. HLA and being used for epitopic prediction for HBV and tuberculosis. ProPred1 allows the user to predict antigenic apitopes for 47 MHC I alleles and ProPred allows epitopes prediction for 51 MHC II alleles. Predictions through these tools can be carried out at various thresholds from 1 to 10%. The algorithms designed for the working of these tools are based on linear coefficients of matrices. Maximum of the matrics were retrieved from BIMASS where the score of each peptide is calculated in multiplication and/or sum up manner. For example the score of following peptide "PACDPGRAA" can be calculated by using following equation:

Score = P(1) × A(2) × C(3) × D(4) × P(5) × G(6) × R(7) × A(8) × A(9)

Score = P(1) + A(2) + C(3) + D(4) + P(5) + G(6) + R(7) + A(8) + A(9)

Where P (1) is score of P at position 1.

Only the promiscuous epitopes with score higher than the chosen threshold score were assigned as predicted epitopes for the selected HLA alleles [30]. For the following study the default threshold i.e. 4% was used where the sensitivity and specificity are nearly the same for most of the HLA alleles available in ProPred1 and ProPred server. Moreover, MHC I alleles were predicted by keeping the proteosome and immunoproteosome filters on at 5% threshold because most of the MHC binders having a proteosomal cleavage site at C-terminal have higher likelihood to be T-cell epitopes [31]. The predicted promiscuous epitopes were positioned in the table in a decreasing order of their score.

### Epitope Conservancy Analysis

All the predicted epitopes of HCV 3a proteins of Pakistani origin were subjected for worldwide conservancy analysis among HCV genotype 1, 2 and 3. 5 sequences against each HCV protein (used for epitope prediction) were retrieved from NCBI randomly. The predicted epitopes of HCV 3a (Pakistani origin) along with 5 selected sequences of individual genotypes (genotype 1, 2 and 3; one at a time) were submitted to epitope conservancy analysis tool available at IEDB database (http://tools.immuneepitope.org/tools/conservancy/iedb_input). All the epitopes having 77-100% conservancy were selected while rejecting the epitopes having variation at the anchor residues. The anchor residues in the predicted epitopes were highlighted by making it bold. The epitopes that were 100% conserved in the selected proteins of the 3 viral genotypes 1, 2 and 3 were also fully bold. Epitopes with 88/77% conservancy were with single or double amino acid variation respectively and to highlight them bold format was used in the conservancy column against each genotype.

Asteric sign (*) indicates that one out of five selected sequences either does not respond to epitope conservancy or have conservancy lower then 77%. Double asteric sign (**) indicates that only one sequence responds for 77-100% conservancy to the selected epitope.

### Validation of varied amino acids using pI value

The Peptides with single or double amino acid variation were analyzed for their hydropathic characteristics or pI value [32]. The pI gives the information that the varied residue retained the amino acid group or diverted from its normal group in a particular peptide under consideration and thus provides information to be used or their rejection. All the varied amino acid residue with diverted group (with considerable change of pI value) were separated from other using superscript "D" for single variation and "DD" for diverted group for doubly varied residues. The superscript "D" in doubly varied residues of particular peptides represents the partial variation i.e. one of the varied residue retained the amino acid group while other residue shifted the amino acid group by a considerable change of pI value.

## Results

HCV 3a genome of Pakistani origin comprises 9474 bp with GC content 2622 and 2700 respectively. The GC contents are 12.35% higher then AT contents. The genome encodes a polyprotein that subsequently get fragmented into structural and non structural protein of obvious molecular weight. The envelope protein E2 comprises highest moleculat weight 38755.3 KDa (Table 1). Leucine (L) a neutral nonpolar amino acid residue has the highest percent of repetition (13.1%) in E2 protein. The least repeated residue of E2 is a basic polar Lysine (K) (1.4%). The shortest segment viral protein is NS4a (5751.69 KDa molecular weight) comprising 54 amino acid residues. Leucine (L) and Valine (V) have highest percentage of repetition (14.8) and Histidine (H), Methionine (M), Threonine (T) and Tryptophan (W) are the least repeated amino acid residues (1.9%). The molecular weight of other viral proteins and percent repetition of their amino acid residue for were listed in Table 1. The percentage of amino acid residues gives an out look for their pI value and their probability of incidence in the antigenic epitopes.

**Table 1:** It comprises the data of HCV genome size, Proteins, Molecular weight and %age of highly repeated and least repeated amino acid residues in individual bases

Bases	**No**.	Proteins	aa Number	**Mol. Wt**.	Highly repeated aa	% of repetition	Least repeated aa	% of repetition
Total bp	9474	Capsid	114	12985.8	R	18.4	C/F	0.9

A	1974	Core	75	7638.88	L	16	E/K/M/Y	1.3

C	2700	E1	190	20643.9	V	11.1	E	1.1

G	2622	E2	350	38755.3	L	13.1	K	1.4

T	2178	NS3	149	15423.6	A/G	11.4	N	0.7

		NS4a	54	5751.69	L/V	14.8	H/M/T/W	1.9
		
		NS4b	194	20167.5	A	13.4	C	0.4
		
		NS5a-1a	62	6700.72	G	14.5	E/D	1.6
		
		NS5a-1b	101	11224.6	P	11.9	K/W	1

F (Phenylalanine), I (Isoleucine), L (Leucine), M (Methionine), V (Valine), W (Tryptophan) and Y (Tyrosine) were mainly the anchor residues for MHC II predicted epitopes and are nonpolar in nature. Total 150 epitopes were predicted against 51 alleles of MHC II (Table 2). The highest number of epitopes was represented by E2 protein comprising 20% of all MHC II predicted epitopes. VFLLNPCGL, FVILVFLLL, WHINSTVLH, FNLLDVPKA, LELINTHGS, VQYLYGVGS are the promiscuous binders of 45-50 MHC II alleles. E2 is followed by NS2 and NS4B proteins representing 14.66% of the predicted MHC II epitopes. In case of NS2 VRAHVLVRL, VILLTSLLY and VRLCMFVRS are the best binders both in term of score and the HLA allele coverage (50-51 MHC II alleles). FFNILGGWV, VNLLPAILS and VVNLLPAIL are the best binders of NS4b protein both in terms of HLA coverage (41 HLA coverage for the first epitope and 51 for the next 2 epitopes) and binding efficiency. LVVGVICAA, FNILGGWVA, WQKLEAFWH, IQYLAGLST and VVGVICAAL are also the epitopes of good quality covering 31 to 35 HLA alleles available in ProPred. For the NS5a_1a only three epitopes (MRLAGPRTC, FISCQKGYK and VVSTRCPCG) were predicted as promiscuous binders with the binding score higher then the selected threshold. Out of these three epitopes MRLAGPRTC is capable of binding all the HLA alleles available in ProPred server while FISCQKGYK and VVSTRCPCG bind 22 and 25 HLA alleles respectively. The predicted promiscuous binders against other proteins were also summarized in table 2.

**Table 2:** Predicted HLA II epitopes HCV Proteins of Pakistani origin and their conservancy in Genotype 1, 2 and 3 worldwide

Epitope start Position	Predicted T-cell epitopes	HLA alleles	HCV genotype 1	HCV Genotype 2	HCV Genotype 3
**Capsid**					

43	**L**GVRATRKA	23	LGVRATRK**T**^D^	LGVRATRK**T**	LGVRATRK**T**^D^

36	**L**PRRGPR**L**G	15	**LPRRGPRLG**	**LPRRGPRLG**	**LPRRGPRLG**

106	**W**GPNDPRRR	16	WGP**T**DPRRR	WGP**T**DPR**H**R^D^	WGPNDPRRR

34	**Y**VLPRRGPR	24	Y**L**LPRRGPR	Y**L**LPRRGPR	YVLPRRGPR

21	**V**KFPGGGQI	8	VKFPGGGQI *	**VKFPGGGQI**	**VKFPGGGQI**

35	**V**LPRRGPRL	9			VLPRRGPRL

45	**V**RATRKASE	25	VRATRK**T**SE^D^	VRATRK**T**SE^D^	VRATRK**T**SE^D^

30	**V**GGV**Y**VLPR	39	VGGVY**L**LPR *	VGGVY**L**LPR	VGGVYVLPR

15	**I**RRPQDVKF	6			IRRPQDVKF

95	**W**LLSPRGSR	28	**WLLSPRGSR**	**WLLSPRGSR**	**WLLSPRGSR**

29	**I**VGGVYVLP	3	IVGGVY**L**LP *	IVGGVY**L**LP	IVGGVYVLP

82	**W**PLYGNEGC	10	**WPLYGNEGC**	**WPLYGNEGC**	**WPLYGNEGC**

85	**Y**GNEGCGWA	11	**YGNEGCGWA**	YGNEGCGWA *	**YGNEGCGWA**

33	**V**YV**L**PRRGP	1	VY**L**LPRRGP	VY**L**LPRRGP	VYVLPRRGP

**Core**					

61	**F**L**L**ALLSCL	50	FLLALLSCL	FLLALLSC**I**	FLLALLSCL

64	**L**ALLSCLIH	45	LALLSCL**TV**^DD^		LALLSCLIH *

15	**F**ADLMGYIP	41	**FADLMGYIP**	**FADLMGYIP**	**FADLMGYIP**

24	**L**VGAPVGGV	44	LVGAP**L**GG**A**		LVGAPVGGV *

63	**LL**ALLSCLI	36	LLALLSCL**T**^D^	LLALLSC**IT**^D^	LLALLSCLI *

62	**FLL**ALLSCL	24	FLLALLSCL	FLLALLSC**I**	FLLALLSCL *

32	**V**ARALAHGV	10		VARALAHGV	VARALAHGV

21	**Y**IPLVGAPV	28	YIPLVGAP**L**	YIP**V**VGAP**L**	YIPLVGAPV

19	**M**GYIPLVGA	26	MGYIPLVGA	MGYIP**V**VGA	MGYIPLVGA

**E1**					

58	**Y**VGATTASI	41			YVGATTASI *

140	**M**VVAHILRL	39			MVVAHILRL*

2	**W**RNTSG**LY**V	27			WRNTSGLYV

138	**V**G**M**VVAHIL	28			

56	**V**K**Y**VGATTA	21			V**R**YVGATTA^D ^*

9	**Y**VLTNARSN	31			YVLTN**DC**SN^DD^

161	**W**GVLAGLA**Y**	15	WGVLAG**M**AY	WGV**VF**GLAY	WG**I**LAGLAY

93	**F**LVGQAFTF	11	FLVGQ**L**FTF		FLVGQAFTF

181	**I**IMVMFSGV	91			IIMVMFSGV

130	**M**MMNWSPAV	35	MMMNWSP**TA**^D^		MMMNWSPA**M**

134	**W**SPA**V**G**M**VV	6			WSPA**M**GMVV *

132	**M**N**W**SPA**V**G**M**	14			MNWSPA**M**GM *

169	**Y**YT**M**QGNWA	18			YY**S**MQGNWA

47	**W**TPMTPTVA	21			WTP**V**TPTVA *

172	**M**QGNWAKVA	25	M**V**GNWAKV**L**^D^	MQG**A**WAKV**I**^D^	WTP**V**TPTVA^D ^*

145	**I**LRLPQTLF	19			ILRLPQTLF

**E2**					

122	**M**LPHHRPVV	3			

151	**V**FLLNPCGL	48			

337	**W**E**F**VILVFL	4			WEF**IV**LVFL

339	**F**VI**L**VFLLL	46			F**IV**LVFLLL

35	**W**HINSTVLH	41			

342	**L**VFLLLADA		L**L**FLLLADA	L**L**FLLLADA	LVFLLLADA

100	**V**LLAYAPRP	50			

198	**F**RPLLPHRL	47			

218	**V**RLGALVDT	12			

62	**F**NLLDVPKA	45			

26	**L**ELINTHGS	46			

57	**F**YYHK**F**NLL	12	FYYHKFN**SS**^D^		FYYHKFN**ST**^DD^

83	**V**GPLDRCQH	26			

58	**Y**YHK**F**NLLD	24			

286	**L**LHSTTELA	17	LLHSTTE**W**A		LLHSTTELA

129	**V**VVGTTDPK	14	VVVGTTD**KL**^DD^	VVVGTTDRL^DD ^*	VVVGTTD**A**K

320	**V**QYLYGVGS	46	VQYLYGVGS		VQYLYGVGS

159	**L**LVVGGLGG	14			

293	**L**AILPCSFT	7			LAILPCSFT

335	**L**KWE**F**VI**L**V	4			LKWEF**IV**LV

322	**Y**LYGVGSGM	5	YLYGVGS**SI**^D^		YLYGVGSGM

300	**F**TPMPALST	17			FTPMPALST

245	**F**YTVQGEDV	4			

18	**I**VRGPEQRL	26			

100	**V**LLAYAPRP	4			

257	**V**WHRFTAAC	19	V**E**HR**L**TAAC^D ^*		

206	**L**LQETSRGH	8			

1	**Y**ITGGTAAR	8			

267	**W**TRGERCDI	10			WTRGERC**E**I

310	**I**HLHQNIVD	11	IHLHQNIVD *		IHLHQNIVD

**NS2**					

101	**V**RAHVLVRL	51			VRAHVLVRL

62	**V**ILLTSLLY	50			VILLTSLLY *

73	**L**VFDIAKLL	24	LVFDI**T**KLL^D ^*	LVFDI**T**KLL^D ^*	L**I**FDI**T**KLL^D^

153	**L**KDLAVATE	7			LKDLAVATE *

113	**F**VRSVTGGK	37			

130	**V**GRWFNTYL	11			VGRWFNTYL *

123	**F**QMAILSVG	31			FQM**I**IL**H**VG^D^

137	**Y**LYDHLAPM	21			YLYDHLAPM

74	**V**FDIAKLLIA	23	VFDI**T**KLL**L**A ^D ^*	VFDI**T**KLL**L**A^D^	

107	**LV**RLCMFVR	36			LVRLCM**L**VR

108	**V**RLCMFVRS	51			VRLCM**L**VRS *

89	**YF**VRAH**V**LV	33			YFVRAHVLV

11	**IL**VLFGFFT	15			

37	**Y**AICRCESA	18		I**IN**GLPVSA^D^	Y**T**ICRCESA^D ^*

33	**W**WNQ**Y**AICR	8			WWNQY**T**ICR^D^

185	**I**LCGLPVSA	10	I**IN**GLPVSA *	I**IN**GLPVSA	ILCGLPVSA

145	**M**QHWAAAGL	18			MQHWAAAGL

50	**V**PPLLARGS	21			VP**S**LLARGS^D ^*

88	**LY**LIQAAIT	35			LYLIQ**T**AIT^D ^*

158	**V**ATEPVIFS	14	VA**V**EPV**V**FS^D^	VA**V**EPV**V**FS	VATEPVIFS

37	**Y**AICRCESA	19			YTICRCESA^D ^*

175	**W**GADTAACG	11	WGADTAACG *	WGADTAACG *	WGADTAACG

**NS3**					

4	**V**QVLSTATQ	46	VQ**IV**STATQ	VQVLS**SV**TQ^D^	

43	**L**QMYTNVDQ	42			

129	**V**CTRGVAKA	21	VCTRGVAKA	VC**A**RGVAK**S**^DD ^*	

24	**W**TVYHGAGS	13	WTVYHGAG**T**	WTVYHGAG**N**	

84	**VI**PARRRGD	18	VIP**V**RRRGD *		

138	**L**Q**F**IPVETL	45			

140	**F**IPVETLST	43	FIPVE**N**L**G**T^D^		

6	**V**LSTATQTF	19	**IV**STATQTF		

53	**L**VGWPAPPG	29	LVGWPAP**Q**G	LVGWP**S**PPG^D^	

27	**Y**HGAGSRTL	22	YHGAG**T**RT**I**		

14	**F**LGTTLGGV	10			

77	**L**VTREADV**I**	25	LVTR**H**ADVI ^D ^*	LVTR**N**ADVI^D ^*	

98	**L**SPRPLACL	12		LSPRPL**ST**L^D^	

124	**IF**RAAVCTR	44			

**NS4a**					

23	**V**VIVGHIEL	43	VVIVG**R**I**I**L^DD^	VVIVG**R**I**V**L^DD^	VVIVGHIEL

3	**W**VLLGG**VL**AA	43	WVL**V**GGVLAA	WVL**V**GGVLAA	WVLLGGVLAA

4	**V**L**L**GG**VL**AAL	40	VL**V**GGVLAAL	VL**V**GGVLAAL	VLLGGVLAAL

38	**V**PDKEVL**Y**Q	11			VPDKEVLYQ *

24	**V**IVGH**I**ELG	8			VIVGHIELG

10	**L**AALAA**Y**CLS	8	LAALAAYCLT *	LAALAAYCLS	LAALAAYCLS

16	**Y**CLSVGC**V**V	6		YCLS**T**GCVV^D^	YCLSVGCVV

26	**V**GHIELGGK	9			VGHIELGGK

25	**I**VGHIELGG	29			IVGHIELGG

20	**V**GCVVIVGH	15			VGCVVIVGH

9	**VL**AALAA**Y**C	9	VLAALAAYC*	**VLAALAAYC**	**VLAALAAYC**

29	**I**ELGGKPA**L**	14			IELGGKPAL

**NS4b**					

81	**FF**NILGGWV	41			FFNILGGWV

153	**V**NLLPAILS	51	**VNLLPAILS**	**VNLLPAILS**	**VNLLPAILS**

152	**VV**NLLPAIL	51			

39	**W**N**F**VSG**I**QY	16	WNF**I**SGIQY	WNF**I**SGIQY	WNFVSGIQY

165	**L**VVGVICAA	35	LVVGV**V**CAA	LVVGV**V**CAA	LVVGVICAA

82	**F**NILGGWVA	32	**FNILGGWVA**	**FNILGGWVA**	**FNILGGWVA**

81	**FF**NILGGWV	5			FFNILGGWV

63	**LM**AFAASVT	9	LMAF**T**AS**I**T^D^	LMAF**T**A**A**VT^DD^	LMAF**T**ASVT^D^

27	**W**QKLEAFWH	35		WQKLE**V**FW**A**^D^	WQKLEAFWH *

167	**V**GVICAALL	11	VGV**V**CAA**I**L	VGV**V**CAA**I**L	VGVICAA**I**L

45	**I**QYLAGLST	35	**IQYLAGLST**	**IQYLAGLST**	**IQYLAGLST**

64	**M**AFAASVTS	23	MAF**T**AS**I**TS	MAF**T**A**A**VTS^DD^	MAF**T**ASVTS^D^

84	**I**LGGWVATH	24	ILGGWVA**AQ**^DD^	ILGGWVA**AQ**^DD^	ILGGWVATH

103	**VV**SGLAGAA	10		V**GA**GLAGAA^D^	VVSGLAGAA

166	**VV**GVICAAL	31	VVGV**V**CAA**I**	VVGV**V**CAA**I**	VGVICAA**I**L

85	**L**GGWVATHL	3	LGGWVA**AQ**L^DD^	LGGWVA**AQ**L^DD^	LGGWVATHL

60	**V**ASLMAFAA	15			VASLMAF**T**A^D^

41	**F**VSG**I**QYLA	8	F**I**SGIQYLA	F**I**SGIQYLA	FVSGIQYLA

139	**F**KIMGGELP	21		FKIM**S**GE**V**P^D^	FKIMGGE**F**P *

9	**L**QRATQQQA	14			LQRATQQQA *

122	**L**DILAGYGA	6			LDILAGYGA *

104	**V**SGLAGAAI	3			VSGLAGAAI

**NS5a_1a**					

39	**M**RLAGPRTC	51	MR**IV**GPRTC *	FISCQKGY**R**^D ^*	MRLAGPRTC*

3	**F**ISCQKGYK	22	F**F**SCQ**R**GYK^DD ^*		FISCQKGYK *

19	**V**VSTRCPCG	25			V**M**STRCPCG *

**NS5a_1b**					

73	**L**LRDEIT**F**V	20	LLRDE**V**TF**Q**^D^*	LLRDE**V**TF**Q **^D ^**	LLRDEITFV *

16	**W**RVAANSYV	33	WRVAA**EE**YV^DD ^*	WRVAA**SE**YV^D^	WRVAANSYV

55	**F**TEVDGVRL	4	FTE**L**DGVRL*	FTEVDGVRL **	FTEVDGVRL

80	**FV**VGLNSYA	25			FVVGLNSYA *

32	**F**HYITGATE	16			FHYITGATE

61	**V**RLHRYAPP	27	VRLHRYAPA*		VRLHRYAPP *

87	**Y**AIGSQLPC	20	Y**VV**GSQLPC *	VRLHRYAP**A**^D ^**	YAIGSQLPC *

23	**YV**EVRRVGD	14	YVEV**T**RVGD^D ^*	YVEV**T**RVGD^D ^**	YVEVRRVGD

**Bold **amino acid residues in T-cell Epitope column indicates the anchor residues

**Bold **individual amino acid residues in HCV Genotype 1, 2 and 3 columns indicated the variation in peptide in comparison to the predicted epitope

*Indicates that one of the protein sequence selected for epitope conservancy either does not respond or have conservancy lower then 70%

** Indicates that only one of the protein sequence from selected sequences respond to epitope conservancy

^D ^Indicates that amino acid residue in case of single/double variation diverted their group compared to primary epitope using pI value

^DD ^Indicates that both amino acid residues in case of double variation diverted their group compared to primary epitope using pI value

Total 69 epitopes were predicted as promiscuous epitopes for MHC I alleles. The anchor residues in case of MHCI are quite varying both in amino acid residues and also in their nature. Mostly represented anchor residues are neutral nonpolar and neutral polar. However, quite small percentage of anchor residues were also acidic polar and basic polar in nature. The highest number of MHC I binding epitopes were represented by NS4b protein comprising 26% of all MHC I predicted epitopes. NFVSGIQYL epitope of NS4b is the best promiscuous binder of highest binding score. NS4b is followed by NS2, E2 and NS3 proteins representing 20.28% (NS2 epitopes) and 11.59% (for E2 and NS3). In case of NS2, 14 promiscuous epitopes were predicted with varying binding efficiency. GSRDGVILL, DGVILLTSL, WAAAGLKDL and LQVWVPPLL are the good binders both in term of score and the HLA allele coverage (21, 28, 27 and 28 alleles respectively). E2 predicted epitopes covers 20 to 28 HLA alleles except the PLLHSTTEL epitope that covers only 11 HLA alleles but with highest binding efficiency. NS3 epitopes covers 8 to 25 HLA alleles and were also ranked on the basis of their binding efficiency predicted by the score. The least represented epitopes were by NS5a_1a. It comprises only one epitope (HVKNGSMRL) as predicted promiscuous binders for 16 MHC I binding alleles. The promiscuous binders of MHC I for other proteins were also predicted and summarized in table 3.

**Table 3:** Predicted HLA I epitopes HCV Proteins of Pakistani origin and their conservancy in Genotype 1, 2 and 3 worldwide

Epitope start Position	Predicted T-cell epitopes	HLA alleles	HCV genotype 1	HCV Genotype 2	HCV Genotype 3
**Capsid**					

38	**R**RGPRLGVR	9	**RRGPRLGVR**	**RRGPRLGVR**	**RRGPRLGVR**

35	**V**LPRRGPRL	25			VLPRRGPRL

**Core**					

55	**P**GCSFSIFL	8	**PGCSFSIFL**	**PGCSFSIFL**	PGCSFSIFL *

41	**R**ALEDGINF	20		R**V**LEDG**V**NF **	RALEDGINF *

7	**V**IDTLTCGF	15	VIDTLTCGF	VIDT**I**TCGF *	VIDTLTCGF *

35	**A**LAHGVRAL	24	ALAHGVR**V**L	ALAHGVRVL	ALAHGVRAL *

24	**L**VGAPVGGV	18	LVGAP**L**GGA *		LVGAPVGGV *

26	**G**APVGGVAR	9	GAP**L**GG**A**AR *	GAP**L**GGVAR	GAPVGGVAR *

**E1**					

135	**S**PAVGMVVA	14			SPA**M**GMVVA *

86	**G**DVCGAVFL	19	GD**L**CG**S**VFL^D^	GDVCGAV**MI**	GD**M**CGAVFL *

144	**H**ILRLPQTL	22			HILRLPQTL *

156	**I**AGAHWGVL	27	IAGAHWGVL		IAGAHWG**I**L

64	**A**SIRGHVDL	25			ASIR**S**HVDL^D^

**E2**					

285	**P**LLHSTTEL	11		PLLHSTTE**W**^D^	PLLHSTTEL

305	**A**LSTGLIHL	25	AL**T**TGLIHL		ALSTGLIHL

227	**C**SFTPMP**A**L	20	CSFT**TL**PAL^D ^*		CSFTPMPAL

71	**Q**QLQAHHFL	27			

157	**C**GLLVVGGL	28			

212	**R**GHIQPVRL	24			

6	**T**AARGGQGL	25			

157	**C**GLLVVGGL	28			

**NS2**					

172	**V**ITWGADTA	6			VITWGADTA *

75	**F**DIAKLLIA	12	FDI**T**KLL**L**A^D^	FDI**T**KLL**L**A^D ^*	FDI**T**KLLIA^D^

70	**Y**PS**L**VFDIA	15			YPSL**I**FDI**T**^DD^

57	**G**SRDGVILL	21	G**G**RD**A**VILL^D ^**	G**G**RD**A**VILL^D ^*	GSRDGVILL

60	**D**GVILLTSL	26			DGVILLTSL *

148	**W**AAAGLKDL	27		WAA**S**GL**R**DL^DD^**	WAAAGLKDL *

50	**V**PPLLARGS	11			VPPLLARGS *

46	**L**QVWVPPLL	28	L**H**VWVPPL**N**^DD^	L**H**VWVPPL**N**^DD ^*	LQVWVPPLL *

117	**V**TGGKYFQM	16			V**V**GGKYFQM^D ^*

65	**L**TSLLYPS**L**	23			LTSLLYPSL

6	**T**LGAGILVL	48			TLGAG**V**LVL *

73	**L**VFDIAKLL	31	LVFDI**T**KLL^D^	LVFDI**T**KLL^D ^*	L**I**FDI**T**KLL^D^

145	**M**QH**W**AAAGL	26			MQHWAAAGL

178	**D**TAACGDIL	21	DTAACGDI**I**	DTAACGDI**I**^D ^*	DTAACGDIL

**NS3**					

119	**G**HVAGIFRA	8	GH**AV**GIFRA *	GHV**V**G**L**FRA *	

27	**Y**HGAGSRTL	14	YHGAG**T**RT**I**	YHGAG**NK**TL^D^	

128	**A**VCTRGVAK	8	AVCTRGVAK	AVCTRGVAK *	

57	**P**APPGAKSL	11	PAP**Q**GA**R**SL^DD ^*	P**S**PPG**T**KSL^DD^	

98	**L**SPRPLACL	25		LSPRPL**ST**L^D^	

95	**A**SL**L**SPRPL	24			

130	**C**TRGVAKAL	20	CTRGVAKA**V**		

7	**L**STATQTFL	24			

**NS4a**					

3	**W**V**L**LGGVLA	11	WVL**V**GGVLA	WVL**V**GGVLA	WVLLGGVLA

23	**V**VIVGHIEL	21	VVIVG**R**I**I**L^DD^	VVIVG**R**I**V**L^DD^	VVIVGHIEL

10	**L**AALAAYCL	24	LAALAAYCL *	LAALAAYCL	LAALAAYCL

5	**L**LGGV**L**AAL	27	L**V**GGVLAAL	L**V**GGVLAAL	LLGGVLAAL

**NS4b**					

96	**P**QSSSAFVV	6			PQSSSAFVV

40	**N**FVSGI**Q**YL	30	NF**I**SGIQYL	NF**I**SGIQYL	NFVSGIQYL

81	**F**FNI**L**GGWV	13			FFNILGGWV

46	**Q**YLAGLSTL	17	**QYLAGLSTL**	**QYLAGLSTL**	**QYLAGLSTL**

102	**FV**VSGLAGA	9		FV**GA**GLAGA^D^	FVVSGLAGA

54	**L**P**G**NPAVAS	14	LPGNPA**I**AS	LPGNPA**I**AS	LPGNPAVAS

161	**S**PGALVVGV	14	**SPGALVVGV**	**SPGALVVGV**	**SPGALVVGV**

141	**I**MGGELPNA	7			IMGGE**F**P**T**A^D ^*

164	**A**LVVGVICA	11	ALVVGV**V**CA	ALVVGV**V**CA	ALVVGVICA

117	**L**GRVLLDIL	22	LG**K**VL**V**DIL^D^*	LG**K**VL**V**DIL^D^	LG**K**VLLDIL^D ^*

59	**A**VASLMAFA	9	A**I**ASLMAF**T**^D^	A**I**ASLMAF**T**^D^	AVASLMAF**T**^D^

152	**V**VNLLPAIL	15			

113	**G**IGLGRVLL	24			GIGLG**K**VLL^D ^*

56	**G**NPAVASLM	12	GNPA**I**ASLM	GNPA**I**ASLM	GNPAVASLM

52	**S**TLPGNPAV	21	STLPGNPA**I**	STLPGNPAV	STLPGNPAV

85	**L**GGWVATHL	26	LGGWVA**AQ**L^DD^	LGGWVA**AQ**L^DD^	LGGWVATHL

145	**E**LPNAEDVV	11			

99	**S**SAFVVSGL	22			SSAFVVSGL

**NS5a_1a**					

33	**H**VKNGSMRL	16	HVKNGSMRI *	HVKNGSMRI **	HVKNGSMRL

**NS5a_1b**					

49	**V**PAAEFFTE	6	VPA**P**EFFTE *	VPA**P**EFFTE **	VPAAEFFTE

79	**T**FVVGLNSY	10	TF**Q**VGLN**Q**Y^D ^*	TF**T**VGLNS**F**^D ^*	TF**T**VGLNSY^D ^*

76	**D**EITFVVGL	19	DE**V**TF**Q**VGL^D ^*	DE**V**TF**T**VGL^D^*	DEITF**M**VGL *

**Bold **amino acid residues in T-cell Epitope column indicates the anchor residues

**Bold **individual amino acid residues in HCV Genotype 1, 2 and 3 columns indicated the variation in peptide in comparison to the predicted epitope

*Indicates that one of the protein sequence selected for epitope conservancy either does not respond or have conservancy lower then 70%

** Indicates that only one of the protein sequence from selected sequences respond to epitope conservancy

^D ^Indicates that amino acid residue in case of single/double variation diverted their group compared to primary epitope using pI value

^DD ^Indicates that both amino acid residues in case of double variation diverted their group compared to primary epitope using pI value

**Figure 1:**
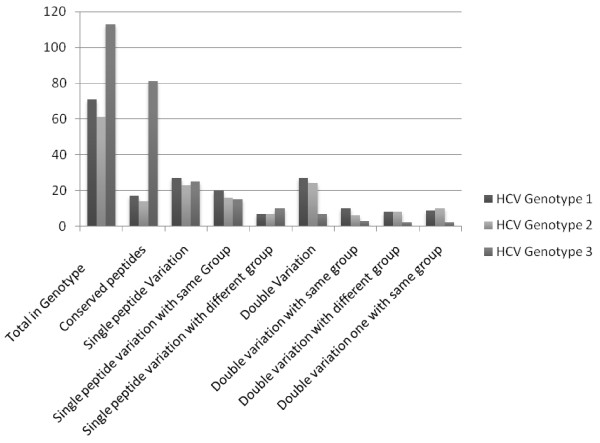
**A comparative analysis of HCV 3a Predictive epitopes against MHC II alleles and their conservancy analysis in Genotype 1, 2 and 3 worldwide**.

Out of total 150 predicted MHC II epitopes, 75.33% were (77-100%) conserve in genotype 3 (Table 1) against the randomly selected viral proteins. Out of 75.33% conserved peptides of genotype 3, 71.68% peptides were 100% conserve while 22.12% peptides were having single residue variation (88% epitope conservancy). Only the 40% peptides of singly varied residues diverted their amino acid group and the pI value while 60% singly varied residues retained the amino acid group as was in the predicted epitope of HCV 3a proteins. 6.19% peptides comprised the 77% epitope conservancy because of double residue variation in the peptides of general population in contrast to predicted epitopes of HCV 3a of Pakistani origin. Out of 6.19%, doubly varied amino acid residues 42.85% peptides retained their amino acid group and nearly same pI value as in case of predicted epitope while 28.57% peptides were having partial group divertion and 28.57% (of doubly varied amino acid residues) peptides diverted their amino acid group because of considerable variation in the pI value. Similar data was also obtained for the HCV genotype 1 and 2 consisting 47.33% and 40.66% conservancy respectively. However, in contrast to genotype 3, only 23.94% predicted epitopes were 100% conserve in randomly selected sequences of genotype 1 and 22.95% in genotype 2. Their rate of single/double residue variation was also predicted and expressed as figure 1.

**Figure 2:**
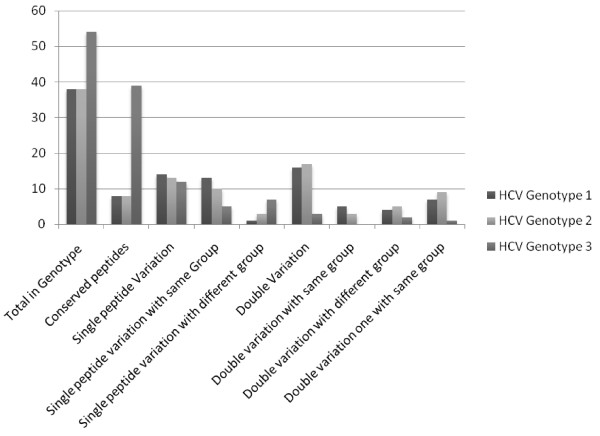
**A comparative analysis of HCV 3a Predictive epitopes predicted against MHC I and their conservancy analysis in Genotype 1, 2 and 3 worldwide**.

Out of total 69 predicted MHC I epitopes, 78.26% were (77-100%) conserve in genotype 3 (Table 2) against the randomly selected viral proteins. Out of 78.26% conserved peptides of genotype 3, 72.22% peptides were 100% conserve while 22.22% peptides were having single residue variation (88% epitope conservancy). 40.66% peptides of singly varied residues retained the amino acid group as was in the predicted epitope of HCV 3a proteins while 58.33% singly varied residues diverted their amino acid group and the pI value. 5.5% peptides comprised the 77% epitope conservancy because of double residue variation in the peptides of general population in contrast to predicted epitopes of HCV 3a of Pakistani origin. Out of 5.5%, doubly varied amino acid residues 66.66% peptides were having partial group divertion and 33.33% (of doubly varied amino acid residues) peptides diverted their amino acid group because of considerable variation in the pI value. Similar data was also obtained for the HCV genotype 1 and 2 consisting 55.07% conservancy. However, in contrast to genotype 3, only 21.05% predicted epitopes were 100% conserve in randomly selected sequences of genotype 1 and 2. Their rate of single/double residue variation was also predicted and expressed as figure 2.

## Discussion

The modern technique for control of HCV infection is a vaccine preparation that can specifically induce antibody-mediated immunity. The rapid advancements in the computational methodologies and immunoinformatics/immuno-bioinformatics provide new strategies for the synthesis of antigen specific epitopic vaccine against infectious agents such as viruses and pathogens. Epitopic vaccine against HIV, malaria and tuberculosis provided promising results and supported the defensive and therapeutic uses of these vaccines [33]. Thus in the present study, a new systematic immunoinformatics approach was applied for the predicted antigenic epitopes of HCV 3a proteins of Pakistani origin followed by diversity and conservancy in other genotypes (1,2 and 3) in randomly selected HCV sequences from NCBI and mainly belong to Thailand, Cuba, UK, USA, China, Japan, France, Italy and Germany. The immunogenic epitopes identified were nanomers and could be used diagnostically to detect HCV specific CTL responses in the patients and after vaccination. A CTL based HCV vaccine might not efficient enough to prevent from infection but it might protect the body from the disease. The analysis showed that the minimal number of epitopes required to represent the complete anigenicity of the whole proteins are significantly smaller then required to represent full length proteins. The majority of the epitopes reported here had intermediate to high HLA binding affinity.

By the use of an efficient CTL based epitope delivery technology; the predicted epitopes could eventually become vaccines in their own or fused as polytopes. The design of the HCV vaccine using conserved epitopes can avoid viral mutation and thus provides more efficient results. The study shows that the predicted epitopes were highly conserved in HCV genotype 3 and also but less conserved in genotype 1 and 2 both for MHC I and MHC II. Moreover, to ensure the viral detection at all stages of its intracellular evolution we have used all the viral proteins. Therefore, the total number of predicted epitopes were also maximized in correspond to the number of covered proteins used for the analysis.

## Abbreviations

HCV: hepatitis C virus; HLA: human leukocyte antigen; MHC: major histocompatability complex; CTL: cytotoxic T lymphocytes.

## Competing interests

The authors declare that they have no competing interests.

## Authors' contributions

AS and SH designed the study. AS performed the immunoinformatics analysis and drafted the manuscript. MI critically reviewed the manuscript. All authors have read and approved the final manuscript.
